# Determination of Thermal DNA‐Stability With Respect to PCR or How to Debunk a Pseudoscientific Claim

**DOI:** 10.1002/bmb.70029

**Published:** 2026-01-21

**Authors:** Vivien Dycks, Andreas Beyer

**Affiliations:** ^1^ Dept. of Molecular Biology, Faculty of Engineering and Natural Sciences Westphalian University of Applied Sciences Gelsenkirchen/Bocholt/Recklinghausen, Campus Recklinghausen Recklinghausen Germany

**Keywords:** PCR, pseudoscience, thermal DNA‐stability

## Abstract

Debunking pseudoscience is difficult, especially for early‐career students and the public. Recently, in particular the Corona pandemic has spawned a whole range of pseudoscientific claims and conspiracy theories, many of which are publicly available in the style of scientific articles on preprint servers, in predatory journals, or in some cases even as regular scientific papers making it difficult to distinguish scientific papers from pseudoscience. One example is a recently published paper claiming that PCR is unreliable because DNA is thermolabile. While experts have the skills to recognize such pseudoscience, inexperienced early‐career students usually have few chances to critically evaluate such claims. Here, we present some simple experiments that combine several aspects: (I) the analysis of a seemingly reputable publication, (II) planning experiments on the physicochemistry of DNA, (III) conducting and analyzing these experiments, and finally (IV) the refutation of the claim that PCR is unreliable. These experiments can be carried out in any standard laboratory with basic molecular biology equipment. They are therefore suitable for undergraduate programs and for high school courses. The model case described here enables students to critically evaluate these claims through practical investigations and to form their own informed opinion. The participants gained new insights into the planning of experiments and a completely new perspective on the subject of science and pseudoscience.

**Abbreviations**
AGE: agarose gel electrophoresis
dstr: double stranded
MW: molecular weight
PCR: polymerase chain reaction
RT: room temperature
sstr: single stranded

## 1 Introduction

Scientific knowledge has expanded dramatically, especially since the end of the 19th century. This applies to both the level of detail and the depth of knowledge. It is therefore no longer possible to gain even a rough overview of the broad scientific landscape: the age of the polymath is over.

As long as there is science, there has also been pseudoscience. Unfortunately, scientific progress has not led to a decline in pseudoscience and conspiracy theories—quite the opposite. Due to the complexity of science and the depth of contemporary scientific knowledge, most people have no chance to critically evaluate the claims of “mainstream science” versus “alternative science” because (a) theoretical knowledge is lacking and (b) pseudoscientific narratives are in most cases even supported by a few scientists. Moreover, pseudo‐scientists are increasingly moving towards publishing their claims in the form of written and open‐access digital resources that have the look and style of scientific papers. They publish it on preprint servers and in predatory journals, where there is limited or no peer review. Now and then they even succeed in publishing their claims in regular scientific journals, as in the example used in this research.

The advantages of preprints are undisputed. But with a global shift in scientific publishing towards an increasing number of preprints and resources where preprints can be deposited, DOI‐assigned, and searched, citing invalid, poorly verified, or incorrect facts can cause harm [1]. The Corona pandemic was an event that has fuelled pseudoscientific activity as well as conspiracy theories and associated narratives. The threat to public health posed by conspiracy theories and pseudoscience is beyond question, as CoViD‐19 has impressively demonstrated [2, 3, 4, 5]. It has been documented that the communication of certain preprints can reach far beyond scientific audiences [6]. Consequently, the risk of spreading false information is even greater when the source is a regular scientific journal.

Because pseudoscientific false claims are usually disregarded by the scientific community for good reason, only a few scientific papers address them directly to refute them. This makes it even more difficult for molecular biology students—and in some cases even graduates—to distinguish “serious” from pseudoscientific papers. So what else can you do but follow your intuition and believe what best fits your own understanding and worldview? [7].

There is a proven didactic principle: it is ideal for a learning process if the relevant facts can be investigated and verified by the learners themselves, for example, by means of a well‐thought‐out and feasible laboratory experiment [8, 9]. Here, we provide an example of a pseudoscientific claim that can be directly tested by undergraduate students or by pupils at the secondary school level. In the wake of the pseudoscientific claims related to SARS‐CoV2/CoViD19, there was a publication (in 2021 as preprint and in 2024 as a regular paper, published by the authors Roberto Serpieri and Fabio Franchi) that claimed that PCR is generally unreliable due to the thermal instability of DNA, calling into question the relevance of any technology involving a PCR reaction as it involves higher temperatures and requires the integrity of the single strands of template and amplicons [10]. There is no need to discuss such unsubstantiated claims, but even students of molecular biology have problems making a differentiated and clear judgment of this item, as we demonstrate here. It is therefore very instructive for learners to check the facts scientifically by conducting an experiment themselves.

Here we present a series of simple experiments we conducted that participants can use to disprove the claims about the stability of DNA themselves and come to their own well‐founded conclusions. The experiments can be carried out in any standard molecular biology laboratory. The set‐up is suitable for both undergraduate (bachelor's) programs as well as for secondary schools if appropriate molecular biology (basic) equipment such as a thermocycler is available.

### 1.1 Learning Objectives

The series of experiments pursues four different didactic objectives at several levels: (I) to train the ability to analyze a scientific (albeit pseudoscientific) publication, (II) to train the planning of scientific experiments, (III) to use these experiments to analyze a specific physico‐chemical property of the biopolymer DNA, and (IV) to expose the pseudoscience behind Serpieri & Franchi's claims about PCR. Section 5.1 describes how these goals are implemented in the (student and pupil) internships. (I) Participants analyze the publication by Serpieri and Franchi [10] to understand the content and how they attempt to substantiate their claim that PCR is unreliable. (II) Undergraduates are asked to try to design experiments in order to verify or disprove this claim (this step is omitted for the pupils). (III) After conducting the experiments—in concrete terms a PCR reaction and a series of denaturation experiments—and then analyzing them, (IV) participants can reflect on their understanding of reliable science and its relationship to pseudoscience.

Since pseudoscience poses a serious threat to knowledge‐, science‐, and technology‐based societies, it should be taken much more seriously in the education system. Learners should be empowered to develop the ability to independently interpret data. With the experiment described here, we want to contribute to this goal.

## 2 Materials and Methods

### 2.1 Materials and Reagents

Plasmid isolation was performed with a standard miniprep kit (Nucleospin Plasmid EasyPure Preparation Kit, Macherey & Nagel).

DNA yield and purity were assessed by UV photometry using a standard UV/vis photometer.

A Biometra thermal cycler (Biometra TRIO 48 Multi Block Thermal Cycler, purchased from Analytik Jena, Germany) was used for incubation of the samples at defined temperatures and well as for PCR.

Restriction enzymes were purchased from New England BioLabs (NEB, www.neb‐online.de).

For PCR, a (standard) Taq polymerase from NEB (catalogue number M0320S) was used. dNTP mix was also purchased from NEB (catalogue number N0447L).

As primers, standard primers were used—M13fw (‐20, modified by one base): 5′‐TGTAAAACGACGGCCAGT‐3′/M13rev (‐27): 5′‐GGAAACAGCTATGACCATG‐3′.

Low‐eeo‐agarose was used for electrophoresis. Staining of DNA in agarose gels was performed with GelRed (Merck, purchased from SigmaAdrich, https://www.sigmaaldrich.com/DE/de/product/mm/sct123 at the recommended concentration of 1:10,000). GelRed is suitable for the detection of both sstr and dstr DNA. The Thermo Scientific O'GeneRuler 1 kb DNA ladder (Fisher Scientific, https://www.fishersci.de/) was used as a molecular weight standard.

Otherwise, standard equipment and reagents were used; no specialized equipment or materials are needed.

#### 2.1.1 Plasmids for PCR and Restriction Digestion

A PCR fragment was generated as starting material for the denaturation experiments (PCR components and PCR profile see below: Section 2.2.2). A cDNA plasmid clone of *Homo sapiens* RNA binding fox‐1 homolog 2 (RBFOX2) orf, transcript variant 1, cDNA (Acc NM_001031695.4), cloned in pDONR211, served as template. To demonstrate the basic effect of thermal cycling, we performed a PCR experiment comparing a cycled sample with an identical non‐cycled aliquot. For this purpose, we used an uncharacterised genomic fragment of the *Escherichia coli* genome that had been cloned into pUC19. This plasmid construct was originally generated during an undergraduate internship in 2019. However, virtually any PCR fragment is suitable for these purposes.

In addition, denaturation experiments (Section 2.2.3) were performed with linearized plasmid DNA (pUC19, 2686 bp).

### 2.2 Methods

#### 2.2.1 Plasmids

Plasmid DNA is used as experimental material. Plasmids were propagated in *E. coli* DH5α in standard medium (LB) at 37°C overnight with aeration and shaking. 1.5 mL culture samples were harvested by centrifugation (10 min in a microcentrifuge at 14,000 rpm = max. g). From these, plasmids were prepared using a standard kit (2.1) according to the manufacturer's instructions.

Restriction digestion of the pUC19 DNA was performed with Hind III under standard reaction conditions.

#### 2.2.2 PCR

The ingredients and the PCR profile are listed in Tables 1 and 2, respectively.

**TABLE 1:** Reagents for PCR.

Ingredient	Final conc. or quantity
H_2_O (A.bidest)	Ad 40 μL (= final vol.)
PCR‐buffer	1×
MgCl_2_	2 mM
4 dNTPs	200 μM each
Oligonuceotide primers M13fw and M13rev (standard)	1 μM each
Taq DNA polymerase	4 U
Template (pre‐diluted to 1 ng/μL)	2 ng

**TABLE 2:** PCR profile.

	T	T	Segment	Cycle profile
1.	3 min	95°C	Initial denaturation	
2.	30 s	95°C	Denaturation	Steps 2–4 = PCR‐cycle; = PCR‐cycle; = PCR‐cycle; 18 iterations
3.	30 s	53°C^a^	Annealing	
4.	2 min	72°C	Elongation	
5.	15 min	72°C	Final DNA synthesis	
6.	∞	10°C	Final storage	

^a^ This is the standard value; we have used prolonged primers annealing at 60°C.

#### 2.2.3 Denaturation Experiments

From the DNA fragments obtained, aliquots were taken and subjected to denaturation at 95°C for different periods of time—in some cases followed by incubation at 75°C for 30 min to allow renaturation (as renaturation depends on diffusion, which is faster at 75°C compared to RT). The incubation function of a multiblock PCR cycler (Biometra TRIO) was used for both denaturation and renaturation.

#### 2.2.4 Agarose Gel Electrophoresis

For (native) agarose gel electrophoresis (AGE) 1% w/v agarose gels (30 mL) were prepared. Electrophoresis buffer was 1× TAE (final concentration: 40 mM tris‐acetate pH 8.0; 1 mM EDTA). After boiling in the microwave oven (900 W), the gel solution was cooled to 50°C. GelRed was then added (final dilution 1:10,000, according to the manufacturer) and the gel was poured. Electrophoresis was performed at a field strength of 5 V/cm at room temperature.

#### 2.2.5 Densitometry

The densitometric measurement was carried out with Octave (https://www.gnu.org/software/octave/). The images of the different gel lanes were saved as separate tif files. Densitograms were generated from these images using the following program commands:

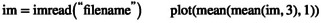




Colors were exchanged using IrfanView64 (https://www.irfanview.com/64bit.htm).

### 2.3 Study Design for Evaluating Learning Effects of the Experiments

#### 2.3.1 Experiments

Participants were given a digest of the review by Serpieri and Franchi [10]. Then they were asked to summarize the content in 5 to 10 sentences. Moreover, they were asked to state what they thought of the claims and whether they believed it—spontaneously and without judgment or grading. After the practical course, they were asked to what extent their opinion on the topic had changed and what they had learned about the topic of science and pseudoscience.

In the internship, two simple experiments, referred to below as (A) and (B), were conducted with the participants (see Section 2.3.2).

(A) a PCR reaction was performed as described (Section 2.2.2); the template was an uncharacterised genomic fragment of the *E. coli* genome in pUC19 (see Section 2.1.1).

(B) 2 μg of pUC19 plasmid were linearized with PstI (1 h at 37°C; reaction buffer and units used as recommended by the manufacturer). Subsequently, six aliquots of 300 ng each were taken from the restriction mixture and subjected to denaturation times between 4 min and 1 h.

#### 2.3.2 Survey

Two cohorts participated in this study:

1. undergraduate students (3rd‐semester university students who had passed their examination in Molecular Genetics; Molecular Biology programme, Westphalian University of Applied Sciences, Recklinghausen, Germany; four internship groups in 2024).
2. secondary school students (pupils attending the upper grades of grammar schools, grade level 11/Q1, aged 16–17 years; two internship groups in 2024 and 2025, respectively).

We consistently refer to these groups as “undergraduates” and “pupils.” When both groups are referred to collectively, the term “participants” is used. Moreover, throughout this manuscript, the terms “learners” and “students” are used in a general sense, without reference to the study or its participants. The internship was structured as follows: (see also Section 3.3)

- The participants were given an excerpt from the publication by Serpieri and Franchi [10] (as German translation). They were also told that the two authors are “real scientists.” Then they were asked for their opinion (not as a standardized survey, but in free‐text form; procedure, see Section 2.3.2)
- Undergraduates were asked to suggest experimental approaches and design an experimental procedure, including controls, to test the thermostability of DNA. This step was omitted for the pupils
- The following experiments were carried out^1^: (1) preparation of plasmid‐DNA, (2) quality and quantity check, (3) restriction digestion to linearize the plasmid, (4) heat incubation of plasmid aliquots (followed by 30 min at 75°C to enable renaturation), (5) in parallel a PCR reaction of the plasmid (an untreated aliquot, diluted to 1 ng/PCR) with modified M13‐primers (see 2.1) was performed and (6) subsequently the samples were analyzed by agarose gel electrophoresis. Then the results were discussed. Finally, the undergraduates had to write up and submit the results in the form of a scientific report (omitted for the pupils).
- A post experimental survey was conducted on the paper published by Serpieri and Franchi.

#### 2.3.3 Ethics Approval Statement

As part of the data collection for this publication, a survey was conducted with participants on six separate internship days, both before and after the experiment. Pupils from two nearby high schools (as part of a school biology practical course) and undergraduates (enrolled in a Molecular Genetics practical course associated with my [A.Beyer's] lecture) were each given an excerpt from a pseudoscientific article about PCR and asked for their opinions. This was followed by a hands‐on practical session in which the claims made in the pseudoscientific text were experimentally tested. Afterwards, participants were asked again for their opinions to assess learning outcomes. The results of this survey are published anonymously in this BAMBED article and any identifying information has been deleted from survey data. The institution at which the study was conducted does not maintain a formal ethics committee, and therefore German research regulations and the standards of good scientific practice (as outlined by the German Research Foundation—DFG) were utilized to determine exempt status as no ethics approval is required for purely anonymous, low‐risk educational research. Based on these criteria, the study was conducted in accordance with all applicable ethical and legal requirements in Germany (Article 4(1) and Recital 26 of the EU General Data Protection Regulation (GDPR), German Research Ethics Guidelines (DFG Code, Guideline 8) and the Data Protection Act of North Rhine‐Westphalia according to which studies involving anonymized, minimally burdensome educational or evaluation data are not subject to approval in Germany, as no personal data is collected or processed [11]). The following conditions applied:

- Participation in the survey was entirely voluntary and based on informed consent of participants.
- No personal or identifiable data were collected, stored, or processed at any point during or after the study.
- The dataset was fully anonymized; no direct or indirect identifiers were retained, and re‐identification of participants is not possible, even with additional external knowledge.
- The study did not involve any sensitive or protected categories of data, such as information relating to health, sexual orientation, ethnicity, political beliefs, or religious affiliation.
- The nature of the research was pedagogical/didactic, focusing on general educational practices, and posed no foreseeable risk to participants.

## 3 Results

### 3.1 PCR Fragment

**FIGURE 1:**
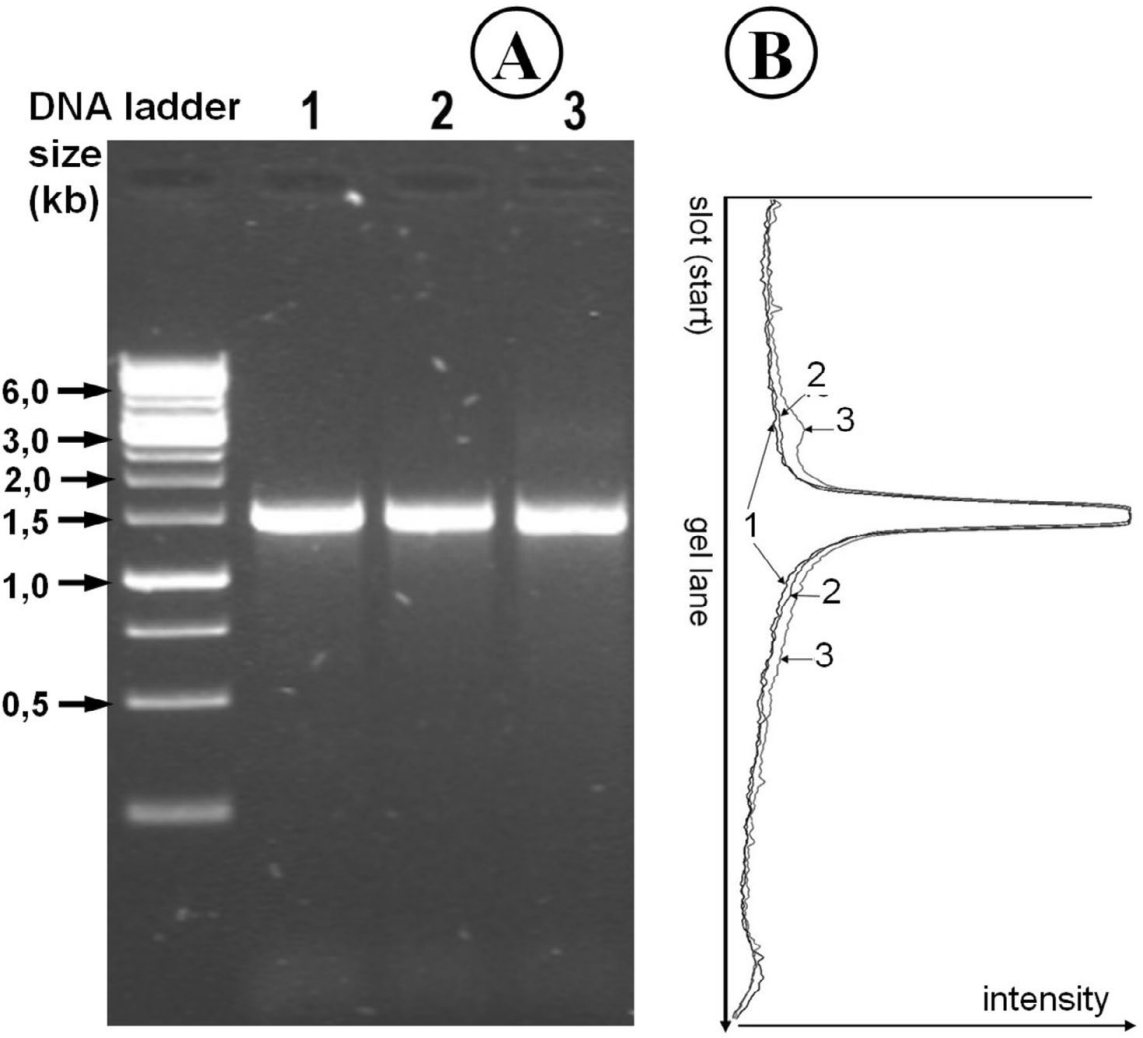
PCR amplification of a cloned cDNA fragment ( *Homo sapiens* RNA binding fox‐1 homolog 2 [RBFOX2] orf, transcript variant 1, Acc NM_001031695.4, cloned in pDONR211) after brief heat treatment, analyzed by agarose gel electrophoresis (AGE). Panel A: DNA samples obtained after PCR were analyzed by AGE using the O'GeneRuler 1 kb DNA ladder as molecular weight standard. The expected size of the PCR fragment is 1.5 kbp. Lanes 1–3 correspond to samples subjected to different denaturation and renaturation conditions: (1) no denaturation after PCR, (2) 95°C for 1 min followed by 75°C for 20 min, and (3) 95°C for 15 min followed by 75°C for 20 min. Each lane contained 300 ng of DNA. The image was slightly overexposed to visualize faint background signals. Panel B: Densitometric profiles of lanes 1–3 from panel A; scans start from the left side below the gel slots.

In order to determine the expected heat‐induced hydrolysis of the DNA, a denaturation experiment (Section 2.2.3) was carried out with the PCR fragment obtained (Section 2.2.2). These samples were finally analyzed with AGE (Section 2.2.4). Figure 1 shows the result.

If the DNA were actually sensitive to heat to a considerable extent, a strong background would be expected even after short denaturation intervals. Figure 1A shows neither a decrease in band intensity nor a significant increase in background in the lanes of the denatured and renatured samples, with the exception of a slight increase in background in sample 3 while the PCR‐band is still clear and sharp. Figure 1B shows the overlay of the three densitograms of the lanes in the gel (Figure 1A), which confirms this result.

### 3.2 Restriction Fragment

**FIGURE 2:**
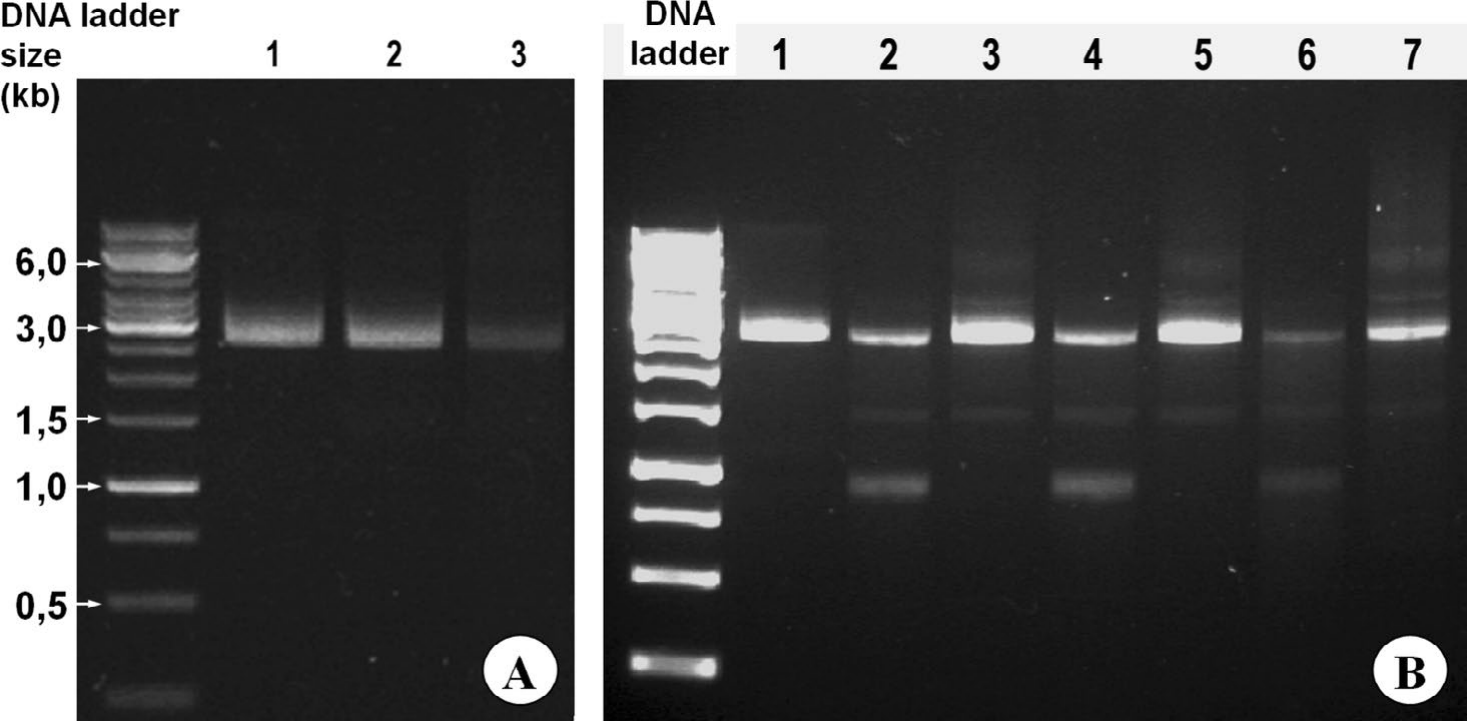
Heat treatment and renaturation of a DNA fragment analyzed by non‐denaturing AGE. Panel A: Linearized pUC19 plasmid DNA was subjected to defined denaturation times at 95°C followed by renaturation at 75°C for 30 min. Lane 1 contained an untreated reference sample. The other samples were denatured for 1 min (lane 2) or 30 min (lane 3). Each lane contained 300 ng of DNA. The expected size of the linearized plasmid is 2.7 kbp. Panel B: Comparison of samples denatured in pairs for 30 s (lanes 2–3), 4 min (lanes 4–5), or 32 min (lanes 6–7). One sample of each pair (namely 2, 4, 6) was directly cooled on ice and the other one (3, 5, 7) was incubated for 30 min at 75°C to allow renaturation. Each lane contained 350 ng of DNA, and the image was overexposed to visualize faint background. DNA size was determined using the O'GeneRuler 1 kb ladder as reference.

The prepared plasmid DNA (Section 2.1.1) had a purity (ratio OD 268/280) of 1.91. It was subjected to a denaturation experiment (Section 2.2.3). Figure 2A shows the results of the different denaturation times (followed by 75°C for 30 min).

After denaturation, the DNA is initially single‐stranded. Longer fragments cannot renature immediately for kinetic reasons. Therefore, a renaturation phase of 30 min was added to facilitate renaturation at increased temperature (and thus higher diffusion rate). The effect of this renaturation step at 75°C was analyzed in the next experiment. For this purpose, the samples were denatured in pairs (Figure 2B: 2/3; 4/5; 6/7) and then treated differently: One sample was immediately cooled on ice until AGE, and the other was incubated at 75°C to allow renaturation. Figure 2B shows the result.

One minute of denaturation has virtually no effect, whereas 30 min of denaturation reduces band intensity and produces a faint background, although the band remains clear and sharp (Figure 2A). Figure 2B shows the effect of denaturation with and without a subsequent renaturation period: only after renaturation at 75°C does a prominent band corresponding to the untreated control sample appear. Moreover, increased background is observed only after prolonged denaturation.

### 3.3 Testing the Experiments With Undergraduates and Pupils

Two simple experiments (Section 2.3.1) were tested with participants (Section 2.3.2).

**FIGURE 3:**
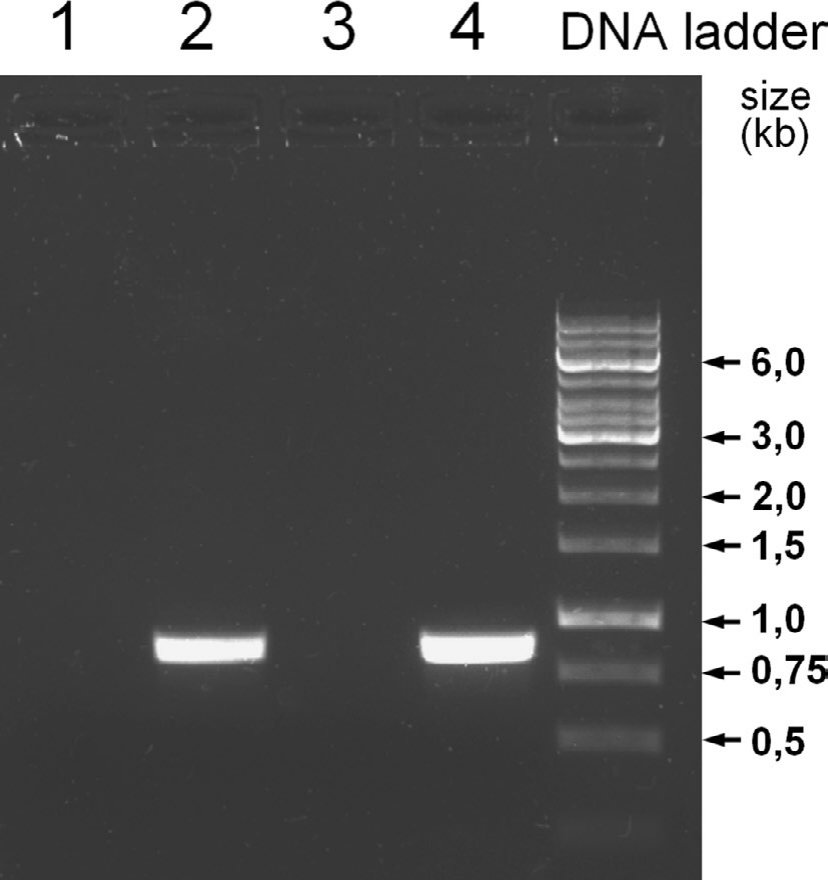
AGE‐analysis of PCR samples with and without cycling. A simple PCR reaction was performed using plasmid DNA—with an uncharacterized genomic fragment of the *E. coli* genome cloned in pUC19—as template (Tables 1 and 2). The expected size of the PCR fragment is 0.8 kbp. The O'GeneRuler 1 kb ladder served as molecular weight marker. Lanes 1–4 represent two PCR samples (duplicates 1 & 2 and 3 & 4). From each reaction, 10 μL were removed before thermocycling (lanes 1 and 3), and the remaining aliquots were analyzed after PCR amplification (lanes 2 and 4).

- A PCR reaction was performed as described (Section 2.2.2). Figure 3 shows the results.

PCR yields a clear, sharp, strong band (lanes 2, 4) whereas 1 ng of input is far too low to produce a visible band (lanes 1, 3).

**FIGURE 4:**
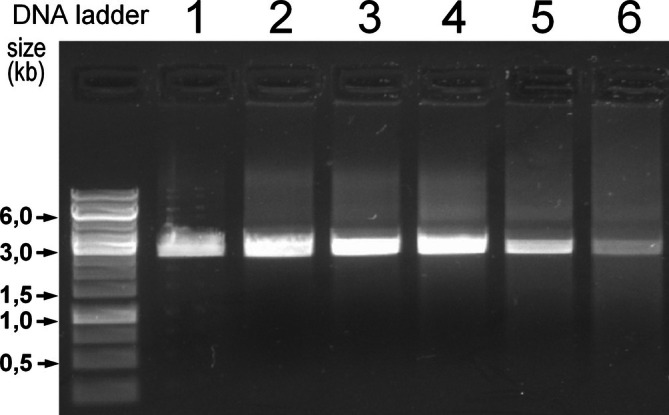
Correlation between denaturation time and the extent of DNA degradation in plasmid samples. Linearized pUC19 plasmid DNA (with an expected size of 2.7 kbp; 300 ng per lane) was heated at 95°C for various durations (lanes 1–6: 0, 4, 7.5, 15, 30, and 60 min, respectively, followed by 30 min at 75°C for renaturation, see 2.2.3) and analyzed by AGE using the O'GeneRuler 1 kb ladder as molecular weight standard. A clear band persisted up to 15 min, while incubation for 30 min caused a moderate decrease in signal intensity. After 1 h of denaturation, only a faint band remained, indicating extensive fragmentation of the DNA. The gel image was slightly overexposed to visualize background smearing.

B Six aliquots of linearized pUC19 plasmid (2 μg each) were subjected to denaturation times between 4 min and 1 h. Figure 4 shows the gel electrophoretic analysis.

Until 15 min denaturation time there is a strong band visible. After 30 min at 95°C, signal strength decreases. After 1 h of denaturation, only a small proportion is still intact.

### 3.4 Didactic Evaluation of the Experiment (3.3)

The results of the questioning (before and after the practical course, see Section 2.3.2) were as follows (Tables 3 and 4).

**TABLE 3:** Results of the questioning before the practical course.

Conclusion/summarizing	Undergraduates	Pupils	Together^a^
Trusts PCR because it is widely used. Criticizes the review by Serpieri and Franchi in a differentiated manner: narrow database, outdated studies, conditions that do not correspond to today's PCR, etc.	7	4	20%
Trusts the PCR because it is widely used, but no specific arguments beyond that	9	6	25%
Believes that PCR works in principle, but based on the paper by Serpieri and Franchi believes (or at least thinks it is possible) that there are problems	3	8	20%
Believes that there are significant problems with the PCR	7	8	25%
Problem not understood	3	2	10%
Total	29	28	100%

*Note*: Each statement it counted (and listed) only once. Primary data excerpts (examples of individual feedback) are provided in the supplement.

^a^ Due to the size of the samples, the percentages were rounded to 5% increments.

**TABLE 4:** Results of the questioning after the practical course.

Conclusion/summarizing	Undergraduates	Pupils	Percentage^a^
Has verbalized in which respect Serpieri and Franchi's criticism is irrelevant.	22	21	80
Has verbalized why the review by Serpieri and Franchi does not meet scientific criteria.	20	13	65
Explicitly emphasizes that the internship has sharpened the view of the topic of science and pseudoscience	15	11	50
Is still of the opinion that the PCR method has considerable problems	—/—	2	5
The statement missed the point	1		[2]
Total^b^	27	25	

*Note*: In this table, the named statements are totaled. This means that a statement may be counted more than once. Primary data (results of the questionings) available in the electronic supplement. The total is less than in Table 3 because two undergraduates did not respond to the 2nd questioning.

^a^ The value refers to the complete cohort, that is, to 54 participants. Due to the size of the samples, the percentages were rounded to 5% increments.

^b^ As some participants either did not provide a second response or did not take part in the internship, the total figures in Tables 3 and 4 are not identical.

## 4 Discussion

The background to this work is the pseudoscientific publication by Serpieri and Franchi that PCR is “not reliable” due to heat‐induced DNA hydrolysis [10]. It is to be seen in connection with pseudo‐scientific criticism of diagnostic PCR systems in corona diagnostics [12].

We have used this publication as a “didactic use case” to show how pseudoscientific claims can be debunked—in this case through rather simple laboratory experiments for students or pupils.

PCR has been one of the most commonly used molecular biology technologies for decades and is therefore ideally suited to show how even well‐established routine laboratory technologies are discussed in pseudoscientific terms and contribute to confusion not only of laypeople. The aim of the paper presented here is to provide a series of experiments that will allow participants to investigate this point for themselves and form their own qualified opinions.

Indeed, all biopolymers are susceptible to hydrolysis, and in an aqueous solution, the susceptibility increases with increasing temperature. However, the question is whether the degree of degradation during PCR is sufficient to say that it “contradicts the claimed paradigm of PCR fidelity and raises the concern that, at least for long sequences, if PCR can amplify some information, such amplified information may be unreliable for diagnostic or forensic applications.” [10].

### 4.1 Interpretation of Results

As can be seen in Figures 1 and 3, a properly performed PCR reaction produces a sharp and well‐defined band. Even a denaturation of a few minutes does not change this. Only a longer incubation at high temperature (95°C for 15 min) leads to a weak background smear (Figure 1A, lane 3; Figure 2). The heat resistance of the DNA—demonstrated by the persistence of sharp bands despite short denaturation—can also be observed with restriction fragments (Figures 2A and 4). The effect of renaturation at 75°C can also be easily detected on a non‐denaturing gel (Figure 2B)—note the presence or absence of a band generated by sstr DNA with an apparent MW of 1 kbp in the samples without and with a renaturation time span, respectively. Even 30 min at 95°C hydrolyzes only about half of the DNA strands and generates a substantial background (lane scan/densitometry not shown). To degrade virtually all DNA strands, more than 1 h at 95°C is required (Figure 4). It should be noted that 30 min at 95°C is longer than the complete denaturation time of even a 45‐cycle PCR.

**FIGURE 5:**
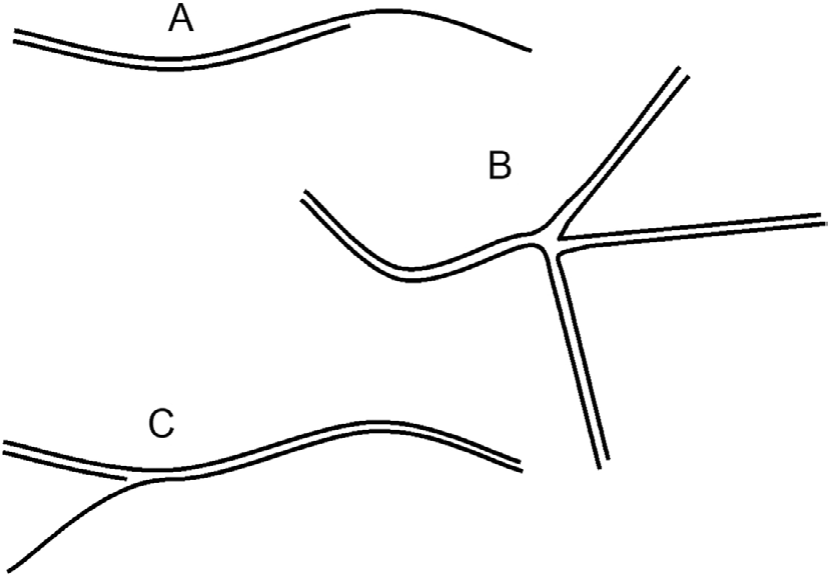
Possible DNA structures formed after heat denaturation and partial hydrolysis. Schematic representations of DNA configurations that can arise during denaturation–renaturation cycles. (A) A complete DNA fragment hybridized with a partially hydrolyzed fragment. (B) A Holliday‐junction‐like structure formed between complementary regions. (C) A complete fragment hybridized both with a partial fragment and another intact fragment, producing complexes of higher apparent molecular weight on non‐denaturing gels.

It must be discussed with the participants where the background with a higher apparent MW than the original fragment could come from (right lane in Figures 1, 3, and 4). Figure 5 shows examples of structures with a higher apparent MW than expected (for intact sstr and dstr DNA fragments).

Taken together, these experiments clearly show what was to be investigated: the thermal stability of DNA is so high that only very small amounts are lost even during the entire PCR process. It takes about 1 h at 95°C to fragment a significant amount of DNA (see Figure 2A, lane 3; Figure 4, lane 6) and this result refutes the claims made by the authors in the paper by Serpieri and Franchi [10].

## 5 Didactics

Is DNA thermostable—at least thermostable enough to ensure a reliable PCR process? Since the 1990s, millions of PCR systems have been established and billions of PCR reactions have been performed, so this question can be considered answered. Even the efficiency parameter can be determined by qPCR [13, 14], and therefore we know that we can achieve (almost) a doubling of amplicons in the exponential phase of the PCR process. However, it should be borne in mind that students and laypersons hardly have the opportunity to carry out such work themselves to prove a concept right or wrong by experimental means.

The experimental workflow for determining the thermostability of DNA, which is presented here, is simple and robust enough to be performed in undergraduate courses or even in high school with appropriate, basic equipment. We have used a standard PCR system and a standard plasmid preparation protocol that yields products of satisfactory purity. In this way, we want to ensure that the experiments work even without high‐purity starting material. We have also avoided all high‐tech equipment (e.g., Sanger sequencing technology and capillary gel electrophoresis) and toxic chemicals such as formamide for denaturing gel electrophoresis. However, certain inconveniences cannot be avoided for this very reason: Only a denaturing gel system based on formamide would completely avoid renaturation and thus provide absolutely clear bands and therefore easy‐to‐interpret results. However, even non‐denaturing AGE is sufficient for the desired learning impact (see below).

### 5.1 Results From the Didactic Evaluation of the Internship

**FIGURE 6:**
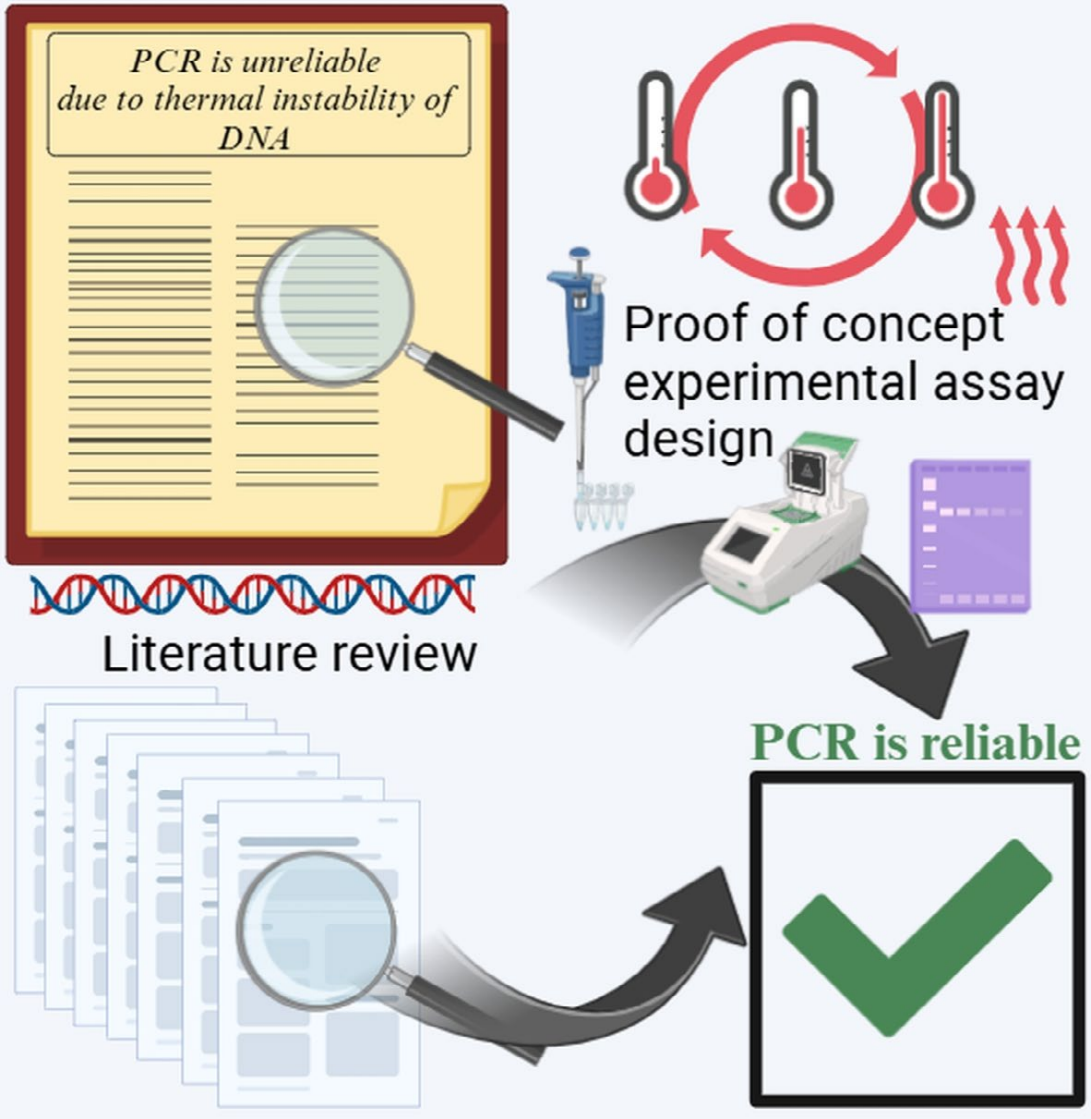
Graphical abstract.

The outcome of the internship explained above (Section 2.3, see also Figure 6) was analyzed. The practical course was conducted with 27 undergraduates and 25 pupils. The results of the individual feedbacks are shown in Tables 3 and 4 (Supporting Information: Feedbacks_translated‐Excerpt.pdf). The preexperimental feedbacks (5.1‐I, 3.4: Table 3; combined data from all participants: undergraduates and pupils): 45% believed that there are indeed problems with PCR. The other 55% argued that PCR obviously works, but only 20% were able to give concrete arguments.

About half of the participating undergraduates had some good ideas for planning the experiments (5.1‐II; data not shown). With the help of guiding questions, they were then able to suggest meaningful experiments in a group discussion. For their reports (5.1‐III), 65% of the undergraduates had researched the backgrounds of the authors Serpieri and Franchi, discovering their links to the coronavirus denialist scene (data not shown).

The result of the second survey (5.1‐II, 3.4: Table 4) was very clear for both undergraduates and pupils. Only a very small percentage were still of the opinion that there are some systematic problems with the PCR method. Fifty percent explicitly emphasized that the internship was helpful with regard to the topic of science vs. pseudoscience. Eighty percent have put into words why Serpieri and Franchi's criticism is irrelevant. All those who had changed their minds agreed that they had mistakenly considered Serpieri and Franchi's publication to be (at least partially) serious and well‐founded. Furthermore, many were dismayed that such pseudoscientific claims exist at all and that they factually cannot be debunked by non‐specialists. Some of the comments included:

We assumed that we could believe what a professor told us or gave us as a text. In retrospect, we realized that we cannot assume that statements made by people with academic titles are always scientifically correct. We asked ourselves why this was never a topic in the lecture. We learned from this to always question statements made by professors and scientists and to look at them critically and not to believe everything, especially since we have knowledge of molecular biology topics through our degree program and should actually know better ourselves.

My personal conclusion from the internship is that, especially with scientific papers, you should look at who exactly is behind the authors, as such pseudo‐scientific papers can look very professional, especially if you are not fully familiar with the subject matter. It can be particularly dangerous when people like corona deniers find such papers and think they are real science and then spread it further. That's why I think it's good that we did this internship, as in this case it shows how easy it is to refute these theses.

I do not believe that these malicious people who rely on people's fears and ignorance can be changed or stopped. The solution should therefore be to make the potential victims immune to such charlatans.

Overall, the internship was rated as instructive, but some participants also criticized the fact that topics such as pseudoscience and conspiracy theories are not really covered in the degree courses and this exercise demonstrates the need for such endeavors.

## Author Contributions

**Vivien Dycks:** most experiments. **Andreas Beyer:** project conception and design, some experiments, draft manuscript preparation. Both authors reviewed the results and approved the final version of the manuscript.

## Funding

Parts of this work were funded by Caramba Chemie GmbH & Co KG, Wanheimer Str. 334, D‐47055 Duisburg. We acknowledge support by the Open Access Publication Fund of the Westphalian University of Applied Sciences.

## Disclosure

This article should be read in continuation of the discussion initiated in Quarterly Reviews of Biophysics. Readers are also referred to the accompanying Q Rev. Biophys. Editor's note, which points out that some claims in the 2024 review by Serpieri and Franchi [10] do not reflect current understanding of PCR DNA amplification and that a detailed critique has since been published (Konrad and Beyer [15]). No generative AI tools were used in the conception, analysis, or interpretation of the work. AI‐based tools were used solely for language editing.

## Conflicts of Interest

The authors declare no conflicts of interest.

## Supporting information


**Data S1:** Feedbacks translated‐Excerpt.

## Data Availability

The data that supports the findings of this study are available in the Supporting Information of this article. Additional material—specifically the full set of all individual feedback—is available upon request.

## Endnotes

^1^
Steps (1) to (3) are optional: they are only required to provide the necessary DNA material. They were conducted in the study course internship. Prepared DNA was provided for internships for pupils.
