# Supplementary material for: Determination of Thermal DNA‐Stability With Respect to PCR or How to Debunk a Pseudoscientific Claim
Source: Biochem Mol Biol Educ. 2026 Jan 21;54(1):49–58. doi: 10.1002/bmb.70029 (PMC12877964; doi:10.1002/bmb.70029)
Supplement: Supplementary file 1 — Data S1: Feedbacks translated‐Excerpt. [file BMB-54-49-s001.pdf]

Results of the questioning

All students and pupils agreed to have their feedback published anonymously. The following table presents four or five selected examples of personal feedback from each practical course, representing the typical range of responses.

The errors in the texts (typos, grammar, punctuation) were corrected manually; the content was not changed. The translation was done using an online program (www.onlinedoctranslator.com).

Each table cell contains individual feedback. left: before the internship / right: after the internship

Study course Molecular Biology April 2024

It is true that most biopolymers - DNA, RNA, proteins, carbohydrate polymers - are sensitive to hydrolysis. In living organisms, biopolymers are hydrolytically broken down by various enzymes due to the low temperatures in the body. Enzymes increase the reaction rate. This hydrolysis enables organisms to break down biopolymers and then utilize them. (...) Starch and glycogen, which are examples of carbohydrate polymers, are broken down into simpler sugars with the help of glycosidases, which in turn serve as an energy source.<br>In PCR, due to the high temperatures during denaturation, hydrolysis can take place without the enzymes that are essential in the body.<br>[Note from the authors Dyks & Beyer: This student did not initially understand what it was all about]	Serpieri and Franchi's criticism of the reproducibility of PCR is not relevant, as they mainly criticize outdated scientific work from the 1950s to the end of the 1980s. The criticized work largely dates from the time before PCR and therefore different information on temperature and time should not be considered as errors in the PCR method.<br>We assumed that we could believe what a professor told us or gave us as a text. In retrospect, we realized that we cannot assume that statements made by people with academic titles are always scientifically correct. We asked ourselves why this was never a topic in the lecture. We learned from this to always question statements made by professors and scientists and to look at them critically and not to believe everything, especially since we have knowledge of molecular biology topics through our degree program and should actually know better ourselves.
While many biopolymers are indeed sensitive to hydrolysis, their degradation often requires specific conditions. These conditions can be elevated temperatures, the presence of active enzymes, or extremely low pH values.<br>In the case of DNA, its degradation mainly involves the disruption of hydrogen bonds between the two strands, resulting in a loss of structural integrity.<br>Regarding criticisms of PCR and concerns about the potential randomness of DNA fragmentation at high temperatures, it is important to recognize that despite its inherent limitations, PCR remains a valuable scientific tool. Scientists know that 100% efficiency is rare in experimental techniques, but as long as the method produces reliable results within the broader scientific context, that's OK.<br>While it is legitimate to explore new denaturation approaches and discuss potential improvements to PCR, such discussions should be based on current research rather than relying solely on studies	This experiment was conducted by molecular biology students and, due to its simplicity, could have been conducted by the critics themselves before writing their paper. However, the critics, Serpieri and Franchi, did not conduct this experiment or any similar experiments that disproved their criticism of PCR. They presumably did so to avoid discrediting Franchi's 2020 book, COVID-19: The Catastrophe Caused by the Virus that Does Not Exist. This book and the criticism of PCR clearly show the intent of their work: to spread misinformation in times like a global pandemic in order to "sell" their ideas. They supported the conspiracy theory that SARS-CoV-2 does not exist, while simultaneously trying to claim and "prove" that the PCR method, which has been widely used for diagnosis during the COVID-19 pandemic, does not work. This ideology was and still is very dangerous for the public. The lack of scientific method and knowledge in their work shows that they are not scientists, but

conducted over 30 years ago.	pseudoscientists. (...)<br>I should definitely question things. To me, this shows that we should have more reliable scientific information accessible everywhere. I do not believe that these malicious people who rely on people's fears and ignorance can be changed or stopped. The solution should therefore be to make the potential victims immune to such charlatans. Not everyone can become a scientist, but it should be common knowledge that claims like "COVID vaccines contain chips for government control" are fake news.
In my opinion, PCR is largely reliable because it is still used today and is indispensable in many areas. Moreover, I know of no other method for quickly replicating genetic material.	I believed Serpiri and Franchi because the report sounded very scientific to me and I have no in-depth knowledge in this area. Pseudoscience can cause problems because it spreads misinformation..
I believe that PCR is still fairly reliable, as it is still used in laboratories for various purposes. If the results were so bad, the method would probably not be used anymore.	My personal conclusion from the internship is that, especially with scientific papers, you should look at who exactly is behind the authors, as such pseudo-scientific papers can look very professional, especially if you are not fully familiar with the subject matter. It can be particularly dangerous when people like corona deniers find such papers and think they are real science and then spread it further.<br>That's why I think it's good that we did this internship, as in this case it shows how easy it is to refute these theses.
I think the assumption that DNA, RNA, proteins and carbohydrate polymers are sensitive to hydrolysis is plausible, as hydrolysis is an important mechanism in living organisms to ensure metabolic reactions, digestive processes and the degradation of biomolecules. The controlled degradation of molecules leads to the maintenance of the organism and therefore a sensitivity to hydrolysis is acceptable. When renowned scientists take this perspective, I tend to trust them, as they are based on extensive research and expertise.	It is important that scientific claims are always critically questioned and supported by solid experimental evidence. The results of the internship clearly show that Serpieri and Franchi's concerns about the PCR method are unfounded and that PCR, as used in practice, is a reliable and precise technique for amplifying DNA.<br>After some research, I would revise my statement about Serpieri and Franchi's work. I realized that a statement that initially seems logical may not always be correct. It would have been possible that a critical attitude and research would be enough to distinguish science from pseudoscience.<br>When I realized that he was a Corona critic, all alarm bells rang. Pseudoscience should be critically analyzed wherever possible in order to expose it.

Study course Molecular Biology August 2024

The authors' results seem conclusive so far. However, it should be remembered that PCR is now widely used, although thermal hydrolysis of DNA was already known at the time of the patent. Therefore, I find it somewhat difficult to believe that PCR is really as unreliable in practice as the authors claim.	Now that I was able to check the reliability of PCR myself during my internship, Serpieri and Franchi's publication seems to me like uninformed talk. Any sources on the reliability of PCR that have been published since its invention are ignored or treated as unreliable without further justification. Therefore, in my opinion, the work should not be considered scientific.
In my opinion, in areas such as medical diagnostics/research and forensic science, methods should be used that are 100% accurate and reliable. Since human life is at stake (be it a diagnosis of a disease or a wrongful conviction in court based on a genetic fingerprint), the result cannot depend on a random break in DNA. Optimizing PCR, while it is possible, to eliminate the problem of DNA degradation could improve medical diagnosis and perhaps eliminate problems that exist today.	The statement by Serpieri and Franchi is misleading. Although it is theoretically correct that PCR can lead to a loss of information due to DNA hydrolysis, this only occurs with longer denaturation times. The results of the practical show that significant DNA hydrolysis can be observed after about half an hour. However, Serpieri and Franchi do not take into account that the denaturation phase in PCR is much shorter. In this short period of time, only minimal DNA hydrolysis of about 1% occurs, which in practice does not lead to any relevant losses. Therefore, their statement is not applicable for practical applications.
The claim that biopolymers are sensitive to hydrolysis sounds quite plausible. It is known that molecules change or lose bonds at certain temperatures (or other extreme conditions). However, I do not understand why PCR is still a widely used scientific analysis method (e.g. in the Corona diagnostic stick). The methodology has been optimized and adapted so that reliable results can be delivered. However, some doubts about PCR remain.	While it is true that DNA can fragment during PCR, the experiment showed that this only occurs after a longer period of time. This contradicts the report which claims that this is a significant problem from the beginning. For me, this shows how important it isto critically question scientific claims and not to believe everything the media reports without checking. Especially when it comes to topics such as epidemics and healing methods, we should be particularly vigilant about how information is presented. This has definitely changed my view of such reports.
The authors' statements do not convince me and I am bothered by their focus on the "golden years of molecular biology". There were certainly many new discoveries at that time by brilliant scientists who drove molecular biology research forward. (...) Since the studies were carried out before the development of PCR, they were not based on the same conditions. For example, Zamenhof, Alexander and Leidy heated the DNA for a whole hour. (...) In order to check the validity of the old studies, one would have to be sure that the conditions generally correspond to those of PCR.	My attitude towards Serpieri and Franchi's publication was already critical before the internship, but was further reinforced by the experiments on the internship day. Their theses are so easy to refute that I have strong doubts that they intended to make a contribution to scientific progress with their paper. Given that both are "corona deniers", I would practically rule out any scientific interest behind their publication. If I found it acceptable to publish pseudo-scientific articles to reinforce doubts about measures to combat the pandemic, I would have chosen a different course of study.
Kary Mullis simply found a way to quickly replicate DNA. He did not take into account the physical or chemical properties of DNA. PCR was patented even though it was known that denaturation could lead to DNA fragmentation. That is why I wonder	With the current findings from the internship, compared to my previous opinion, it can be said that PCR is a valid method for DNA amplification. Before the internship, after reading the publication by Serpieri and Franchi, I had questions myself: "Is

whether PCR is still a suitable method and whether all previous research using PCR is still valid or questionable.	PCR even a suitable method?" I can very well imagine that many people who do not deal with the topic ask themselves such questions. That is why I think it is questionable whether something like this should be published. That is not to say that methods should not be criticized! Nevertheless, one should not rely on "old" papers and publications, especially given the progress that has taken place in the last few decades.

Study course Molecular Biology October 2024

I find the authors' statements about the sensitivity of biopolymers to hydrolysis understandable. I wonder whether there are strategies to control this degradation. It could be exciting to learn more about mechanisms that increase the stability of these molecules. Overall, I find the discussion very stimulating and agree with the statements made by Serpieri and Franchi.	After the internship, I think that the work of Serpieri and Franchi represents an interesting but clearly exaggerated criticism of PCR. The hypothesis that denaturation leads to fragmentation and thus to unreliable results is clearly refuted by our experiments. DNA shows remarkable stability under typical PCR conditions and fragmentation only occurs under extreme conditions (96°C for 90 minutes).<br>The internship helped me personally to better understand the mechanisms of PCR and to evaluate criticism of it and test it experimentally. The practical experience with denaturation times and their analysis by gel electrophoresis was particularly valuable. This enabled me to develop a better understanding of how to generate sound data and test insufficiently supported hypotheses.<br>The internship was therefore very helpful and educational. Even though another topic would certainly have been just as exciting, this topic was excellent for gaining insight into scientific rigor and methodological validity.
Since we have already discussed various hydrolysis reactions in the lecture, this point is not unknown to me. For this reason, I can understand the arguments of the authors. The fact that these authors, who are well-respected in their field, make such statements gives the whole thing additional weight. Overall, they seem to be on a solid scientific basis.	After the experiments during my internship, I gained an important insight: not all publications, even if they were written by scientists, are automatically credible. It is essential to critically examine scientific papers and, where possible, to do your own research before accepting their statements.<br>The paper by Serpieri and Franchi that we analyzed seemed to me more like an attempt to sow doubt or uncertainty where it is not justified. The internship helped me understand for the first time how important it is to have a well-founded, objective review of scientific statements - regardless of the author's reputation.
Temperature certainly has an impact on the accuracy of PCR, but I don't think it makes a significant difference. Unnecessarily high temperatures, however, are still an avoidable risk.	My opinion of the paper by Serpieri & Franchi remains critical. I learned that pseudoscientists also publish scientific papers and don't just spread their nonsense on some dubious platforms. I generally thought the internship was good, it was nice to have carried out this PCR myself.
It is of course thought-provoking that a method as established as PCR should contain fundamental errors. Personally, I don't think there is much to it. After all, PCR is used in many areas where it is effective. I think it is fairly certain that a strand will break here and there due to the heat, but that will definitely not be significant. Practice speaks against this. The paper also repeats itself quite a lot and does not really provide any new evidence that points to a	I didn't find the results of the internship particularly surprising, but I would still say that it was a good experiment for an internship day in the second semester. Dealing with alleged pseudoscience, especially in the early semesters, gives a completely different perspective on what you have learned.<br>I would never think of questioning the PCR, you just learn it all from lectures and accept it as it is. But it could also be that there is actually something to it. In

serious problem. In summary: I would continue to trust PCR.	other words, it is also good to know why such statements are nonsense. In any case, I am of the opinion that you should talk to everyone on an equal footing and take their concerns seriously. Then it is easier to refute nonsensical theories.
In my opinion, this is a very important topic - especially since the PCR method is widespread and has been and is used often (e.g. during the pandemic to detect the Corona virus). I would not be surprised if the PCR is unreliable, but I do not have enough understanding of the whole topic to be able to judge that.	I said at the beginning that I don't know enough about the subject and I still don't really know much about it. I found it surprising that Serpieri and Franchi weren't really right, even though their paper seemed very credible and serious. Someone with little knowledge would believe all of that. I thought it was good that we did the PCR. On the subject of pseudoscience, I definitely learned that you should question everything, even if something seems very serious and comes from scientists.

Study course Molecular Biology November 2024

First of all, Serpieri and Franchi's statement sounds logical because I know that DNA can be broken down by hydrolysis, for example. However, the statement that DNA is much more susceptible to damage/changes or similar doesn't make sense. I'm not sure.	After the internship, I was a little surprised that Serpieri and Franchi were right, but only after a much longer warm-up period than they claim. With regard to pseudoscience, I learned how important clear criticism is for scientific work. I personally found the internship and the protocol very helpful, as it encouraged me to think critically and question things. The topic itself was interesting, but I found Serpieri and Franchi's article somewhat annoying, as many things were repeated.
Personally, I find the statements of the two scientists questionable. The studies used in the paper seem serious, but I find it questionable that for a method introduced in 1985 they only use studies that were carried out 20 to 30 years earlier. It seems as if they are simply keen to confirm their opinion without "thinking further". (...) If you take a closer look at the two authors, I notice that neither of them works in the natural sciences. Roberto Serpieri works in the social sciences department and Fabio Franchi in information technology, which further reduces their credibility.	I found the paper by Serpieri and Franchi strange and not very credible from the start, and this was reinforced by the internship, as the thesis has now been actually tested and refuted. In general, I have learned to be even more skeptical about pseudoscience and to question/check the sources and authors more closely. The internship showed that a professor title does not necessarily stand for competence and seriousness. The internship was very helpful for the problem of pseudoscience, to make people aware of how dangerous pseudoscience is. The paper by Serpieri and Franchi was well suited to this, the experiments in the laboratory were interesting and yet easy to carry out for beginners, and there was a good and up-to-date database with which to write the protocol.
While I find the article interesting and it may indeed raise many questions, I still think that PCR is reliable, especially in the range of methods used today (qPCR etc.). In addition, controls are also used to check the process. The article doesn't seem to me to be coming from a completely neutral point of view. The studies are very old, and hardly any counterarguments are presented or refuted (but that could be because I don't know the whole article). In the end, the whole thing seemed a bit like chatter to me.	During the internship, the paper quickly turned out to be pseudoscientific because it was easy to refute. The internship showed how important it is to question things critically and to check carefully whether claims are really well-founded. It was definitely helpful in learning how to recognize pseudoscience. There are certainly topics that have more practical relevance for the course, but it was still exciting and the level of difficulty was just right.
The claim that biopolymers are sensitive to hydrolysis seems quite logical to me, because even under physiological conditions these molecules are exposed to enzymatic influences that cause their hydrolytic degradation. For example, DNA in cells is constantly damaged, but the organism has repair mechanisms to prevent the accumulation of errors.	The paper by Serpieri and Franchi can serve as an example of how hypotheses based on incomplete or outdated data can lead to conclusions that are not supported by practical experiments. Pseudoscience is often based on unreliable claims or faulty interpretations of data. In this case, despite what the article says, the actual reliability of PCR might indicate that, for example, the experimental conditions are different from those in the cited studies. The article can be useful for understanding how scientific hypotheses can be tested and refuted, as well as emphasizing the importance of empirical data and replication of experiments. I find the

	internship helpful because PCR is a commonly used method, both during our studies and in our future work.

High School course biology December 2024
Joseph-König-Gymnasium, D-45721 Haltern am See

In my opinion, there is definitely a problem with PCR, as it is quite obvious that heat denaturation above 80°C can cause DNA breaks. This means that important, perhaps even crucial, information is lost, which is essential for solving a crime or taking decisive medical measures, for example. For this reason, I believe that there is a problem with PCR.	After having carried out the PCR process myself, I have found that my initial opinion was wrong, because it turns out that DNA only breaks after a very long time due to heat denaturation. Since the denaturation in PCR is much shorter, it can be assumed that important information in the DNA is not lost due to heat denaturation. However, attention must still be paid to the duration of this heat denaturation, because DNA breaks occur after a long time.<br>With regard to science and pseudoscience, I have learned the following: You should definitely always check whether the person who publishes scientific articles is also specialized in this subject area or not. Likewise, when quoting, you should be very careful to see whether these are experts.
I don't think there is a problem with PCR. Studies do show that the intense heating can cause breaks or damage to the DNA, but these problems either only occur rarely or have now largely been resolved by making adjustments to the enzymes or solutions in the PCR. In general, the process must work well in many areas of application, as it is still used. The damage to the DNA should probably only be a real problem in a few isolated cases and areas of application.	I think that PCR is of great importance for science and medicine, as it enables the duplication of individual DNA segments and thus their analysis. It is always useful in various fields of application (eg virus tests or paternity tests) and will probably find even more applications in the future.<br>With regard to publications on scientific issues, I realized that these too should be questioned. Even with scientific texts, there is obviously the possibility that incorrect or outdated information is published. It is therefore necessary that scientific publications are also checked and that readers check whether such a check has taken place or whether they themselves check them using other sources of information before believing them.
The statements in the study by the two university professors on fragmentation in relation to PCR seem to be conclusive overall. The many criticisms and results of various experiments with DNA justify the fact that DNA is unsuitable as an investigation method under certain conditions. This is due, among other things, to the structural and chemical properties of DNA. It can therefore be concluded that there is a problem with PCR in that the method is not 100% reliable. The reasons given in the study show the central problem.	We were able to verify Serpieri and Franchi's assumption that PCR is not reliable in the laboratory ourselves. Gel electrophoresis showed that clear bands were visible despite the DNA being heated for a longer period of time. DNA fragmentation was only evident after heating for more than 30 minutes. Since the denaturation step of PCR takes less time, it can be said that DNA is hardly fragmented during PCR. Serpieri and Franchi have therefore published disinformation in a scientific journal and can be described as pseudoscientists. Since the risk of disinformation and publications by pseudoscientists still exists, accurate scientific work is essential in order to avoid misinformation and to continue to make scientific progress.<br>What I particularly liked about the internship was that we were able to carry out practical experiments

	ourselves and find out how biological processes work and how scientists go about proving and supporting hypotheses. We also learned that as a laboratory technician you need a lot of patience, discipline and precision to achieve precise test results. That wasn't always the case for us students, but in the end it wasn't a problem.
Based on the findings of the two professors' research, it is clear that the PCR method has problems due to heat denaturation, which affects properties of DNA such as viscosity or transformation capacity. This calls the reliability of the method into question. However, the question also arises as to whether the loss of some properties is even significant when PCR only involves doubling short sections.	The two Italian researchers questioned the reliability of PCR; the reason was heat denaturation. However, our experiments, in which we heated DNA for different lengths of time (or not at all: reference), showed that the test results are independent of the heating.<br>It is therefore clear that not every scientific work can be trusted, since, for example, a one-sided consideration/citation of certain results may ultimately lead to incorrect conclusions.
In my opinion, the only problem with PCR is if it is not done precisely or if the DNA is heated too much. In general, I would still consider PCR to be a good method, but care should be taken to set the temperature correctly so that only the hydrogen bonds are broken.	Through the internship and these findings, I learned that PCR works and that PCR is only unusable if the DNA has been heated for too long or too much, which does not happen with PCR. Therefore, PCR normally works (as in the internship) as long as you don't make any mistakes.<br>Through the internship, I learned that one should always be cautious with "scientific" articles and always check the statements in these articles for pseudoscientific statements. Although Serpieri and Franchi's statements about PCR initially sounded very convincing, after closer examination of the statements and sources, one could see that the content did not make sense in the context and the article therefore falls under pseudoscience.
The PCR method is fraught with potential problems due to the need to denature DNA. This denaturation, i.e. the heating step in the PCR method, can lead to fragmentation of the DNA molecules. Studies from the 1950s proved this, for example, through the change in the consistency of the DNA when heated to over 90 degrees, as is the case with the PCR method.	After carrying out the tests to verify the PCR method (more precisely: heat denaturation), we were able to determine that the claims made by Serpieri and Franchi are false. The PCR method - as it is carried out - is perfectly reasonable and works perfectly.<br>The fragmentation of the DNA fragments only takes place after the DNA has been exposed to a much longer temperature than the temperature required in the PCR method.<br>This result also shows us that we should be critical of publications on scientific issues and that questioning the published data can also lead to other findings.

High School course biology January 2025, Adalbert-Stifter-Gymnasium D-44575 Castrop-Rauxel

I believe that PCR is reliable. The article has legitimate criticisms, but they are very imprecise. It does not go into detail about the duration of denaturation. This is now precisely defined, which is why PCR is reliable.	My opinion has not changed after the internship. I see even more now that the criticism in the review is completely unfounded
The publication of the review by Roberta Serpieri and Fabio Franchi refers to some problems with the PCR method, which are based on studies from the 1950s and 1960s. These studies were the first basic principles of the PCR method known today and were partly formulated imprecisely, which caused problems. (...)	It turns out that the research results from 1950/1960 are now outdated, which is why the PCR method can be described as reliable.
I am of the opinion that the publication should be viewed critically, as the results are based on studies from the 1950s/1960s and a lot has changed since then and some of the studies are outdated.<br>I don't think there are any major problems with PCR, as this method has been used for a long time and its reliability has been confirmed by many positive practical experiences, especially in times of coronavirus. In addition, PCR takes place in several steps, so care is taken to ensure that there is not too much fragmentation of the DNA.	The experiment carried out in the laboratory clearly shows that PCR does not damage DNA. The different times for denaturing DNA show that DNA is only destroyed after a period of more than 30 minutes. Since DNA is not denatured for such a long time during PCR, the DNA is hardly damaged, which contradicts the study by Serpieri and Franchi.
In general, the reliability of PCR depends on the way it is carried out. In practice, many of the problems described in the text can perhaps be minimized by careful implementation. The frequent use of PCR in a wide variety of areas shows the reliable results of PCR.	Many of the criticisms mentioned in the paper are outdated and do not reflect the current state of knowledge. One should always be critical of such papers and, if necessary, falsify or verify the claims through experiments, as was done in the internship.
This article presents PCR as an inadequate, non-functional method, citing outdated studies from the 1950s as the basis. These contain inaccurate information that is reflected in the article. For example, the heating time is missing, which, if the length is incorrect, actually leads to DNA fragmentation.	In practice at the moment, PCR is an absolutely reliable method that has helped a lot in crises like Corona and is also used every day by criminologists to solve crimes. The article is of poor quality and not suitable for today's times.
